# Sex differences in the association between cognitive function and 24-hour activity patterns in older adults: a compositional data analysis

**DOI:** 10.3389/fragi.2025.1686847

**Published:** 2025-09-24

**Authors:** Dan Li, Mi Zhou, Xiaomei Song

**Affiliations:** ^1^ Follow‐up Office, The Second Affiliated Hospital of Soochow University, Suzhou, Jiangsu, China; ^2^ Allied Health and Human Performance, Adelaide University, Adelaide, SA, Australia; ^3^ Department of Nursing, The Second Affiliated Hospital of Soochow University, Suzhou, Jiangsu, China

**Keywords:** 24-h activity behaviours, compositional data analysis, physical activity, sedentary behaviour, sleep, sex differences, accelerometry

## Abstract

**Background:**

Cognitive decline is prevalent among older adults and may be associated with their daily activity behaviours. However, no studies have examined how cognitive decline affects older adults’ activity behaviours within a 24-h framework. This study investigates the relationship between cognitive function and 24-h activity behaviours in older adults, further exploring whether these associations differ by sex.

**Method:**

This study analyses data from the eighth wave of the Survey of Health, Ageing and Retirement in Europe, conducting a cross-sectional analysis of 814 older adults. Cognitive function was assessed using the SHARE-Cog tool, encompassing 10-word immediate recall, 10-word delayed recall, verbal fluency, and self-reported memory. 24-h activity behaviours (moderate-to-vigorous physical activity [MVPA], light physical activity [LPA], sedentary behaviour [SB], and sleep) were objectively measured with thigh-worn accelerometers. Compositional multivariate linear regression models were constructed using compositional data as the response variable, with cognitive function measures as predictors.

**Results:**

Higher MVPA was linked to better cognitive outcomes (verbal fluency, 10-word immediate recall, and 10-word delayed recall) while SB and longer sleep related to poorer performance, with these associations being stronger in women (model p ≤ 0.001). Among women, cognitive outcomes were significantly associated with all activity behaviours (p range = 0.010–0.045). Women who self-reported poor memory and scored 0 on the verbal fluency spent approximately 45% of their day in SB, whereas those reporting excellent memory and scoring 60 spent 40.06% (37.18%, 42.86%) and 36.41% (31.53%, 41.10%) of their day sedentary, respectively. In contrast, men’s 24-h activity composition did not vary significantly with cognitive function (p range = 0.051–0.845).

**Conclusion:**

Older adults with better cognitive function tend to engage in more PA and reduce sedentary and sleep time. This relationship differed by sex, with females’ activity behaviours being more sensitive to cognitive function changes.

**Implications:**

These findings suggest that interventions promoting healthy lifestyles in older adults should account for cognitive function, particularly in females.

## Introduction

With advancing age, individuals often experienced varying degrees of cognitive decline, including memory loss and diminished executive function (Murman, 2015). Such decline not merely impaired older adults’ ability to live independently, quality of life, and social engagement but also increased demand for medical and caregiving resources (Saraçlı et al., 2015; Song et al., 2023). Older adults with cognitive decline typically required more caregiving support, frequent medical interventions, and greater family and societal resources (Culberson et al., 2023; MacLeod et al., 2021; Welch et al., 2022). This intensified the healthcare burden in an increasingly aging global population.

Prior studies showed that reduced physical activity (PA), increased sedentary behaviour (SB), and sleep disturbances heightened risks of cardiovascular disease, metabolic disorders, musculoskeletal decline, and mental health issues (Sánchez-Sánchez et al., 2024; Tian et al., 2023; Wang et al., 2021; Zuo and Wu, 2022). Older adults with cognitive impairment often exhibited these unhealthy behaviour patterns more than their healthier peers. Those with cognitive impairment spent more time sedentary and less in PA, increasing their health risks (Hartman et al., 2018; Olanrewaju et al., 2022). However, most studies have primarily relied on self-reported questionnaires, such as the International Physical Activity Questionnaire (IPAQ), to assess daily activity behaviours. These approaches are limited by recall bias and often fail to capture the full 24-h activity cycle. In addition, most studies analysed PA, SB, and sleep separately, ignoring their interrelationships within a 24-h framework. Such isolated approaches risked introducing misleading correlations and collinearity issues. In reality, PA, SB, and sleep are interdependent, where an increase in one reduces time for another. Thus, analysing cognitive function’s relationship with a single behaviour in isolation could misrepresent activity patterns in cognitively impaired individuals. There remains a significant gap in comprehensively examining 24-h activity behaviour patterns across older adults with varying cognitive functions.

Sex differences warrant explicit investigation because the determinants and manifestations of both cognitive ageing and 24-h activity behaviours are sex-specific. Biologically, the hormonal milieu across the life course (e.g., oestrogen decline in menopause), neurovascular risk profiles, and body composition may alter how sedentary time, MVPA, and sleep relate to cognition. Social and behavioural factors, such as gendered roles, caregiving demands, and work patterns—shape time allocation across the 24-h composition, yielding distinct activity and sleep profiles. This suggests the relationship between cognitive function and activity behaviours may differed by sex. Testing sex as an effect modifier therefore enables more accurate inference and supports targeted, sex-responsive prevention and intervention strategies. Yet, no studies systematically investigated this sex difference.

To address these gaps, our study examines the association between cognitive function and older adults’ 24-h activity behaviours within a compositional framework. The use of thigh-worn accelerometers provides objective and continuous measurement, offering a more accurate and integrated representation of daily activity behaviours while also reducing collinearity issues. The null hypothesis is there are no association between cognitive function and older adults’ 24-h activity behaviours, and no sex difference in this relationship.

## Methods

### Study design

This study utilizing a cross-sectional analysis with secondary data. The data used is from the Survey of Health, Ageing and Retirement in Europe (SHARE), a database consisting of samples of community-based adults aged 50 years or older (Börsch-Supan et al., 2013). The initial data collection was carried out in 2004, with subsequent data waves collected at biennial intervals. The present study utilized accelerometer and cognitive function data from wave 8, involving 46,733 participants from 27 countries, which is the only wave containing the necessary accelerometry data. All participants included were anonymous. The data collection commenced in October 2019 and was suspended in March 2020 due to the outbreak of COVID-19 (Bergmann and Börsch-Supan, 2021). Use of the SHARE data (8th wave) was approved by the Ethics Committee of the Max Planck Society for the Progress of Science. SHARE-ERIC’s activities related to human subjects research are guided by international research ethics principles such as the Respect Code of Practice for Socio-Economic Research and the Declaration of Helsinki. The report of this study followed the STROBE guideline (Cuschieri, 2019).

### Measures

#### 24h accelerometer data

Accelerometer data were collected from a subsample in ten European countries: Belgium, Czech Republic, Denmark, France, Germany, Italy, Poland, Slovenia, Spain, Sweden. The participants were instructed to wear a triaxial accelerometer (Axivity AX3, Axivity Ltd., Newcastle upon Tyne, United Kingdom) on their upper thigh for eight consecutive days, both day and night (Bergmann and Börsch-Supan, 2021). Participants could choose either the left or right thigh according to their own preference. The Axivity AX3 is a valid instrument, and its reliability and validity have been confirmed in older adult populations (Clarke et al., 2017; Hedayatrad et al., 2020). The accelerometers were set to a sampling frequency of 50 Hz (with a range of ± 8 g). The raw accelerometer data were processed at SHARE central using ActiPASS Version 1.61beta, an open-source software based on the Acti4 algorithm for posture and activity recognition in data obtained from thigh-worn accelerometers (Skotte et al., 2014). The algorithms from ActiPASS were then used to identify 11 activities, including NonWear, Lie, Sit, Stand, Move, Walk, Run, Stair, Cycle, Other, Sleep, and LieStill. The time allocated to “LieStill”, “Sit” or “Lie” was considered as SB. The time allocated to “Stand”, “Move”, “Walk Slow” (walking with a cadence lower than 100 steps/min), “Other” without any periodic movements, and “Other” with periodic movements at a cadence lower than 100 steps/min was classified as light physical activity (LPA). Moderate-to-vigorous physical activity (MVPA) was defined as the time allocated to “Run”, “Cycle”, “Stair”, “Walk” with a cadence above 100 steps/min, or “Other” activities with periodic movements at a cadence ≥100 steps/min. Additionally, sleep duration was determined by the time allocated to the “sleep” activity. Only participants with at least 4 days of data and 16 h of wear each day were included in the analyses.

#### Cognitive function

SHARE Cognitive Instrument (SHARE-Cog) is a new, short cognitive screening instrument developed and validated to assess cognition in the SHARE. In this cross-sectional analysis, it has good–excellent diagnostic accuracy for identifying cognitive impairment (O’Donovan et al., 2023). SHARE-Cog contains three subtests/domains: 10-word registration; verbal fluency, and 10-word recall.

For the 10-word registration task, participants were read a list of 10 words (randomly selected from four possible lists) and asked to immediately recall as many as possible. Before the task, they were told that the list was purposely long and would not be repeated. Only one attempt was allowed. Scores ranged from 0 to 10, based on the number of words recalled. In the verbal fluency task, participants named as many animals as possible in 1 minute. If silent for 15 s, they were prompted to continue. Any real or mythical animal names were accepted, including breeds and sex/age variations, while repetitions and proper nouns were excluded. The score ranged from 0 up to 100. The 10-word recall task followed verbal fluency, where participants were asked to recall any words from the original list: “A little while ago, the computer read you a list of words… Please tell me any of the words you can remember now.” Scores again ranged from 0 to 10, based on the number of words recalled.

Self-perceived memory reflects an individual’s self-assessment of their current memory ability. The question on self-perceived memory was adopted from Health and Retirement Study, with good reliability and validity in European older adults (Hajek and König, 2016; Ofstedal et al., 2005; Wallace and Herzog, 1995). Participants rate their memory on a five-point Likert scale: Excellent, Very Good, Good, Fair, or Poor. This subjective measure is widely used in epidemiological and aging research to assess memory.

### Statistical analysis

Only complete case were included for analysis. Demographic characteristics were summarized as means ± standard errors (M ± SE) for continuous variables and as percentages for categorical variables. Time spent in each activity behaviour was reported using compositional means.

Compositional multivariate linear regression models were used to examine associations between cognitive function and 24-h activity behaviours. Time-use components for each activity behaviours were transformed into isometric log-ratio (ilr) coordinates and modelled as response variables, with four cognitive function measures (10-word registration and recall, verbal fluency test, self-perceived memory) entered as predictors in separate models. All models were adjusted for sex, age, country, years of education, and body mass index. Regression results are presented as coefficients with 95% confidence intervals [95% CI]. Separate models were also conducted for male and female subgroups.

To aid interpretation, estimated marginal means of 24-h activity behaviours were calculated for each unit increase in cognitive function, based on the adjusted models. Statistical significance was set at *p* < 0.05. All analyses were conducted using the R package compositions (Van den Boogaart and Tolosana-Delgado, 2013).

## Results

### Descriptive statistics

Table 1 presents an overview of the included and excluded participants’ demographic characteristics, time allocation across activity behaviours, and cognitive function indicators. The participant flowchart is provided in Supplementary Figure S1. Baseline characteristics were comparable between included and excluded participants. Sex-stratified demographic characteristics are provided in Supplementary Table S1. A total of 814 individuals were included in the analysis, comprising 41.0% males and 59.0% females, with a mean age of 68.9 years. Participants were drawn from multiple countries, including Poland (15.0%), Germany (13.5%), and Denmark (4.4%). In terms of educational attainment, 59.3% had completed Year 12 or below, 24.1% held a diploma, and 16.6% had a university degree or higher.

**TABLE 1:** Descriptive statistics results.

Variable name	Levels	Stats	
		Included (N = 814)	Excluded (N = 41)
Demographic characteristics			
Sex	Female	480 (59.0%)	22 (53.7%)
	Male	334 (41.0%)	19 (46.3%)
BMI	Mean ± SD	27.5 ± 4.9	28.3 ± 5.1
Age	Mean ± SD	68.9 ± 8.9	69.0 ± 9.3
Education level	Year 12 or below	483 (59.3%)	27 (65.9%)
	Diploma	196 (24.1%)	4 (9.8%)
	University or above	135 (16.6%)	10 (24.4%)
Country	Belgium	76 (9.3%)	4 (9.8%)
	Czech Republic	103 (12.7%)	3 (7.3%)
	Denmark	36 (4.4%)	1 (2.4%)
	France	75 (9.2%)	5 (12.2%)
	Germany	110 (13.5%)	7 (17.1%)
	Italy	66 (8.1%)	1 (2.4%)
	Poland	122 (15.0%)	4 (9.8%)
	Slovenia	97 (11.9%)	2 (4.9%)
	Spain	66 (8.1%)	5 (12.2%)
	Sweden	63 (7.7%)	9 (22.0%)
24 h activity behaviour (Mins)			
MVPA	Mean ± SD	53.3 ± 30.6 (4%)	53.8 ± 40.5
LPA	Mean ± SD	283.0 ± 110.8 (21%)	243.9 ± 118.2
SB	Mean ± SD	572.3 ± 125.0 (43%)	519.6 ± 149.3
Sleep	Mean ± SD	431.5 ± 99.0 (32%)	463.6 ± 122.9
Cognitive function			
Self-perceived memory	Poor	26 (3.2%)	0
	Fair	134 (16.5%)	7 (17.1%)
	Good	436 (53.6%)	22 (53.7%)
	Very good	194 (23.8%)	9 (22.0%)
	Excellent	24 (2.9%)	3 (7.3%)
10-word registration memory	Mean ± SD	5.6 ± 1.6	5.3 ± 1.6
10-word recall memory	Mean ± SD	4.2 ± 2.1	4.1 ± 2.4
Verbal fluency test	Mean ± SD	22.0 ± 7.1	21.9 ± 7.5

BMI, body mass index; MVPA, Moderate-to-vigorous physical activity; LPA, light physical activity; SB, sedentary behaviour.

On average, participants spent 572.3 ± 125.0 min per day in SB, accounting for 43% of their daily time. This was followed by sleep at 431.5 ± 99.0 min (32%) and LPA at 283.0 ± 110.8 min (21%). Time spent in MVPA was relatively low, averaging 53.3 ± 30.6 min (4%).

Regarding cognitive measures, the majority of participants rated their self-perceived memory as “Good” (53.6%), followed by “Very good” (23.8%) and “Fair” (16.5%). Fewer participants rated their memory as “Poor” (3.2%) or “Excellent” (2.9%). The mean score for the 10-word registration task was 5.6 (SD = 1.6), while the mean for the 10-word recall task was 4.2 (SD = 2.1). The average score on the verbal fluency test was 22.0 (SD = 7.1).

### The association between cognitive function and 24h activity behaviour

Associations between cognitive function and 24-h activity behaviour were examined using compositional data analysis (Table 2). Overall model p-values indicated statistically significant relationships between activity behaviours and verbal fluency, 10-word registration, and 10-word recall memory (p < 0.001), but not for self-perceived memory (p = 0.095). Specifically, higher verbal fluency scores were significantly associated with a greater proportion of time in MVPA and less time in SB and sleep relative to the remaining behaviours. Both 10-word registration and recall scores were positively associated with MVPA and negatively associated with SB and sleep. No significant associations were observed between any indicators and LPA.

**TABLE 2:** Associations of cognitive function and accelerometer-measured 24-h activity behaviours.

Variable	MVPA			LPA			SB			Sleep			Model P value
	Beta_Ilr1_	95%CI	P	Beta_Ilr1_	95%CI	P	Beta_Ilr1_	95%CI	P	Beta_Ilr1_	95%CI	P	
Self-perceived memory	−0.04	[−0.11, 0.03]	0.262	0.02	[−0.02, 0.05]	0.397	−0.01	[−0.05, 0.03]	0.679	0.03	[−0.01, 0.07]	0.095	0.095
10-word registration memory	0.05	[0.01, 0.09]	0.009	0.01	[−0.01, 0.03]	0.201	−0.03	[−0.05, 0.00]	0.019	−0.04	[−0.06, −0.02]	<0.001	<0.001
10-word recall memory	0.04	[0.01, 0.08]	0.004	0.01	[−0.01, 0.02]	0.306	−0.02	[−0.04, 0.00]	0.012	−0.03	[−0.05, −0.01]	<0.001	<0.001
Verbal fluency test	0.02	[0.01, 0.03]	<0.001	0	[−0.01, 0.00]	0.246	−0.01	[−0.01, 0.00]	<0.001	−0.01	[−0.01, 0.00]	0.004	<0.001

MVPA, Moderate-to-vigorous physical activity; LPA, light physical activity; SB, sedentary behaviour.

The estimated changes in activity behaviours associated with changes in cognitive function are illustrated in Supplementary Figure S1 and Supplementary Table S2. Individuals with “Excellent” self-perceived memory spent 3% (≈43.2 min) less time in SB per day compared to those with “Poor” memory (Poor: 48.80% [42.43%, 54.62%]; Excellent: 45.92% [40.58%, 50.96%]) (Supplementary Figure S2a), while their sleep time increased by 3% (Poor: 31.63% [30.62%, 32.54%]; Excellent: 34.52% [33.65%, 35.31%]) (Supplementary Figure S2a; Supplementary Table S2). However, physical activity levels (both LPA and MVPA) did not vary with changes in self-perceived memory (Supplementary Figure S2a). For 10-word recall test, participants with higher scores spent more time in both LPA and MVPA, and less time in SB and sleep (Supplementary Figures S2b,c). Finally, as shown in Supplementary Figure S2d, higher scores on the verbal fluency test were associated with increased time in MVPA but less sedentary time. Participants scoring 0 on the test spent, 1.6% (≈23 min) of the day in MVPA and 49% (≈706 min) in SB, whereas those scoring 60 spent over 6% (≥≈86 min) in MVPA and only 42% (≈605 min) in SB (Supplementary Table S2). Sleep time remained consistent across verbal fluency levels.

### The association between cognitive function and 24h activity behaviour across sex

Table 3 presents the associations between cognitive function and 24-h activity behaviours stratified by sex. Among males, the overall model *p*-values indicated no statistically significant associations between 24-h activity behaviours and any cognitive function domain. In contrast, for females, the overall models showed significant associations across all cognitive function measures. Specifically, higher verbal fluency scores were positively associated with time spent in MVPA (*p* = 0.006) and negatively associated with SB (*p* = 0.020) and sleep (*p* = 0.042). Higher delayed recall scores were not associated with MVPA, SB, and sleep at the pre-defined alpha level of 0.05.

**TABLE 3:** The association between cognitive function and 24 h activity behaviour across sex.

Sex	Variable	MVPA			LPA			SB			Sleep			Model P value
		Beta_Ilr1_	95%CI	P	Beta_Ilr1_	95%CI	P	Beta_Ilr1_	95%CI	P	Beta_Ilr1_	95%CI	P	
Male	Self-perceived memory	−0.03	[-0.13, 0.06]	0.481	0.03	[-0.02, 0.07]	0.268	−0.03	[-0.08, 0.03]	0.337	0.04	[-0.01, 0.08]	0.147	0.845
	10-word registration memory	0.07	[0.02, 0.12]	0.011	0.01	[-0.02, 0.03]	0.445	−0.03	[-0.06, 0.00]	0.025	−0.04	[-0.07, −0.02]	0.001	0.332
	10-word recall memory	0.04	[0.00, 0.08]	0.038	0.01	[-0.01, 0.03]	0.269	−0.02	[-0.05, 0.00]	0.064	−0.03	[-0.05, −0.01]	0.002	0.250
	Verbal fluency test	0.02	[0.01, 0.03]	0.001	0.00	[-0.01, 0.00]	0.19	−0.01	[-0.02, 0.00]	0.004	−0.01	[-0.01, 0.00]	0.075	0.051
Female	Self-perceived memory	−0.05	[-0.17, 0.06]	0.379	0.00	[-0.07, 0.07]	0.962	0.03	[-0.04, 0.09]	0.444	0.03	[-0.05, 0.10]	0.481	0.045
	10-word registration memory	0.03	[-0.03, 0.09]	0.333	0.02	[-0.02, 0.05]	0.311	−0.01	[-0.05, 0.02]	0.376	−0.03	[-0.07, 0.00]	0.075	0.013
	10-word recall memory	0.05	[0.00, 0.09]	0.066	0.00	[-0.02, 0.03]	0.772	−0.02	[-0.05, 0.00]	0.093	−0.03	[-0.06, 0.00]	0.074	0.019
	Verbal fluency test	0.02	[0.01, 0.03]	0.006	0.00	[-0.01, 0.01]	0.72	−0.01	[-0.02, 0.00]	0.020	−0.01	[-0.02, 0.00]	0.042	0.010

MVPA, Moderate-to-vigorous physical activity; LPA, light physical activity; SB, sedentary behaviour.

Supplementary Figure S3 illustrates the estimated changes in 24-h activity behaviours associated with cognitive function across sex. Overall, females spent less time sleeping and more time in LPA compared to males, while time spent in SB and MVPA was comparable between sexes. As self-perceived memory improved, women showed a notable reduction in SB (Poor: 44.97% [41.75%, 48.06%]; Excellent: 40.06% [37.18%, 42.86%]), and increases in both sleep (Poor: 32.04% [31.57%, 32.47%]; Excellent:35.73% [35.26%, 36.17%]), and LPA (Poor: 19.84% [17.91%, 21.90%]; Excellent: 21.46% [19.61%, 23.40%]) (Supplementary Figure S3a; Supplementary Table S3). No visual variation in activity behaviours was observed across 10-word registration scores for either sex (Supplementary Figure S3b). In the 10-word recall test, women with higher scores engaged in more LPA and MVPA, and less SB and sleep, compared to those with lower scores (Supplementary Figure S3c). Women scoring 0 on the test spent 2% (≈29 min) of the day in MVPA, 44% (≈634 min) in SB, and 37% (≈533 min) in sleep; the corresponding proportions among women scoring 10 were 4% (≈58 min), 40% (576 min), and 31% (≈446 min), respectively (Supplementary Table S3). A similar trend was observed in men, though their sedentary time remained relatively unchanged (Supplementary Figure S3c). For the verbal fluency test (Supplementary Figure S3d), higher scores were associated with reduced sedentary time and increased PA in both sexes, with these associations more pronounced among women.

## Discussion

This study found that older adults’ cognitive function was associated with their 24-h activity behaviours. Activity patterns varied across different levels of cognitive function. Generally, older adults with higher cognitive function were observed to spend less time sedentary and more time in PA, especially MVPA. This relationship was different by sex. Females’ 24-h activity behaviours were more strongly associated with cognitive function, while males’ cognitive function showed little association with activity patterns.

### Comparison with previous studies

Several studies systematically explored the link between older adults’ 24-h activity behaviours and cognitive function, most treating cognitive function as the dependent variable and highlighting PA’s role in enhancing cognition (Collins et al., 2025; Dumuid et al., 2022; Fanning et al., 2017; Lai et al., 2024; Wei et al., 2021). For example, A Taiwanese isotemporal substitution study reported that replacing sedentary or sleep time with more LPA improved cognitive function (Lai et al., 2024). Similar findings from Australia noted that overall cognitive and executive function in older adults was linked to 24-h activity, with more MVPA associated with better cognition (Dumuid et al., 2022). Using cognitive function as the independent variable, this study confirmed this association from a reverse perspective, aligning with prior findings and further extend current knowledge boundary. Older adults with poorer cognitive function exhibited lower PA levels for several reasons. First, memory decline may have hindered their ability to follow daily plans, such as forgetting scheduled walks or exercise sessions. Additionally, limited comprehension and memory likely reduced their ability to learn and sustain new activities, such as exercise routines or equipment use. Verbal fluency, another cognitive component in this study, assessed vocabulary retrieval and indirectly reflected prefrontal lobe functions like cognitive flexibility, information retrieval speed, and proactive control (Shao et al., 2014). Previous studies linked lower verbal fluency scores to executive function decline, indicating challenges in goal-setting, planning, and behaviour adjustment (Amunts et al., 2020; Whiteside et al., 2016). These abilities were critical for maintaining regular PA, especially without external prompts in daily life (Daly et al., 2015). Individuals with poorer cognitive function may have felt greater psychological burden and uncertainty, leading to more passive behaviours, such as prolonged sitting or avoiding outdoor activities (Balbim et al., 2024).

No prior studies explored sex’s role in the link between cognitive function and 24-h activity behaviours. This study filled this gap, finding that females’ cognitive function was significantly associated with their activity behaviours, unlike males. A plausible explanation is the postmenopausal decline in estrogen: estrogen supports hippocampal and prefrontal circuitry underpinning executive functions (planning, set-shifting, inhibition) that help initiate and sustain physical activity, so reductions may tighten the coupling between cognitive capacity and activity in older women (Shanmugan and Epperson, 2014; Spencer et al., 2008). Estrogen also modulates dopaminergic reward and perceived effort, while menopause-related sleep and somatic symptoms can raise the cognitive/physical “cost” of being active, further strengthening this link (Ennour-Idrissi et al., 2015; Copeland, 2004). By contrast, men experience a more gradual androgen decline, which may attenuate such associations (Ennour-Idrissi et al., 2015; Oliver et al., 2022). Another explanation is that this sex difference may stem from distinct daily behaviour patterns. Older females often managed fragmented tasks requiring strong memory and executive skills (e.g., shopping, cooking, caregiving, or community activities). Thus, cognitive decline significantly disrupted their daily lives, making females more aware of these changes (Oliver et al., 2022). Strong cognitive function enhanced females’ self-efficacy and confidence, which are key predictors of PA participation (El-Sayed et al., 2024; Seeman et al., 1996; Sunarti et al., 2024). Conversely, cognitive decline undermined this confidence, leading to more passive activity choices. Additionally, females experienced faster cognitive decline than males, potentially amplifying perceived cognitive losses and self-doubt (Iso-Markku et al., 2024; Levine et al., 2021). Males, with slower and less severe cognitive decline, perceived less impact from these changes (Oliver et al., 2022).

### Implications

Females’ cognitive function is significantly linked with their 24-h activity behaviours. For females with declining cognitive function or cognitive disorders, their activity patterns warrant close attention. Targeted interventions should aim to reduce SB and increase PA time, particularly MVPA.

### Strengths and limitations

This study’s strength lies in being the first to examine 24-h activity behaviours across older adults with varying cognitive functions. It also pioneered investigating sex difference between cognitive function and activity behaviours, addressing a gap in the literature.

However, the study has limitations. First, despite its multi-center design, it was limited to Europe, lacking data from other regions. Second, the sample size of approximately 800 participants may have limited statistical power and the robustness of findings. The cross-sectional design hindered definitive causal conclusions, and future longitudinal studies could clarify causality. Lastly, cognitive function measurements focused on memory, learning, and verbal fluency, omitting comprehensive assessment of other domains like executive function. Future studies should employ broader cognitive assessment tools to fully explore this relationship.

### Future research directions

Future studies should use larger samples and longitudinal designs to investigate the causal relationship between cognitive function and older adults’ 24-h activity behaviours. The sex difference identified here warrants deeper exploration of its biopsychosocial mechanisms. Studies should also include diverse populations and additional cognitive dimensions (e.g., attention) to comprehensively assess cognitive function’s impact on activity patterns. Moreover, targeted interventions should be developed for high-risk groups with weaker cognitive function, especially females, to promote PA and reduce sedentary time.

## Conclusion

This study systematically examined differences in 24-h activity behaviours among older adults with varying cognitive functions and the sex difference. Results showed that individuals with higher cognitive function engaged in MVPA and less SB, particularly among females. The findings highlight cognitive function is associated with older adults’ daily activity patterns. Public health interventions should prioritize cognitive status, especially in females with cognitive decline, and develop personalized strategies to promote activity, delay functional decline, and improve quality of life.

## Data Availability

The original contributions presented in the study are included in the article/Supplementary Material, further inquiries can be directed to the corresponding authors.

## Abbreviations

BMI, Body mass index; ILR, Isometric log ratio; MVPA, Moderate-to-vigorous physical activity; LPA, Light physical activity; SB, Sedentary behaviour; CoDA, Compositional Data Analysis; SHARE, Survey of Health, Aging and Retirement in Europe.
