# Supplementary material for: Sex differences in the association between cognitive function and 24-hour activity patterns in older adults: a compositional data analysis
Source: Front Aging. 2025 Sep 24;6:1686847. doi: 10.3389/fragi.2025.1686847 (PMC12504469; doi:10.3389/fragi.2025.1686847)
Supplement: Supplementary file 1 [file Supplementaryfile1.docx]

**Supplemental file 1**

**Table S1 Participants flowchart across gender**

| **Variable name** | **Levels** | **Stats** | |
| --- | --- | --- | --- |
|  |  | **Female (N=480)** | **Male (N=334)** |
| **Demographic characteristics** | | | |
| **Age** | Mean ± SD | 68.0 ± 9.1 | 70.2 ± 8.6 |
| **BMI** | Mean ± SD | 27.6 ± 5.3 | 27.5 ± 4.3 |
| **Education level** | ≤12 years | 301 (62.7%) | 182 (54.5%) |
|  | Diploma | 112 (23.3%) | 84 (25.1%) |
|  | University or above | 67 (14%) | 68 (20.4%) |
| **Country** | Belgium | 45 (9.4%) | 31 (9.3%) |
|  | Czech Republic | 67 (14%) | 36 (10.8%) |
|  | Denmark | 18 (3.8%) | 18 (5.4%) |
|  | France | 52 (10.8%) | 23 (6.9%) |
|  | Germany | 61 (12.7%) | 49 (14.7%) |
|  | Italy | 37 (7.7%) | 29 (8.7%) |
|  | Poland | 71 (14.8%) | 51 (15.3%) |
|  | Slovenia | 62 (12.9%) | 35 (10.5%) |
|  | Spain | 38 (7.9%) | 28 (8.4%) |
|  | Sweden | 29 (6%) | 34 (10.2%) |
| **24h activity behaviour (Mins)** | | | |
| **MVPA** | Mean ± SD | 54.3 ± 27.7 | 51.8 ± 34.3 |
| **LPA** | Mean ± SD | 301.9 ± 110.1 | 255.8 ± 106.3 |
| **SB** | Mean ± SD | 550.2 ± 121.1 | 604.0 ± 123.9 |
| **Sleep** | Mean ± SD | 439.6 ± 93.5 | 419.7 ± 105.3 |
| **Cognitive function** | | | |
| **Self-perceived memory** | Poor | 20 (4.2%) | 6 (1.8%) |
|  | Fair | 81 (16.9%) | 53 (15.9%) |
|  | Good | 270 (56.2%) | 166 (49.7%) |
|  | Very good | 94 (19.6%) | 100 (29.9%) |
|  | Excellent | 15 (3.1%) | 9 (2.7%) |
| **10-word registration memory** | Mean ± SD | 5.7 ± 1.7 | 5.3 ± 1.6 |
| **10-word recall memory** | Mean ± SD | 4.5 ± 2.1 | 3.8 ± 2.0 |
| **Verbal fluency test** | Mean ± SD | 22.3 ± 7.3 | 21.6 ± 6.9 |

**Table S2 Adjusted predictions for 24h activity behaviour**

| **Self-perceived memory** | | | | |
| --- | --- | --- | --- | --- |
| **Level** | **MVPA (95%CI)** | **LPA (95%CI)** | **SB (95%CI)** | **Sleep (95%CI)** |
| Poor | 2.87% (2.24%, 3.65%) | 16.70% (13.48%, 20.40%) | 48.80% (42.43%, 54.62%) | 31.63% (30.62%, 32.54%) |
| Fair | 2.74% (2.25%, 3.34%) | 16.82% (14.17%, 19.78%) | 48.09% (42.96%, 52.88%) | 32.35% (31.53%, 33.10%) |
| Good | 2.63% (2.21%, 3.11%) | 16.94% (14.63%, 19.48%) | 47.37% (42.94%, 51.56%) | 33.07% (32.36%, 33.72%) |
| Very good | 2.51% (2.11%, 2.99%) | 17.05% (14.70%, 19.64%) | 46.65% (42.14%, 50.92%) | 33.79% (33.07%, 34.46%) |
| Excellent | 2.40% (1.95%, 2.94%) | 17.15% (14.40%, 20.25%) | 45.92% (40.58%, 50.96%) | 34.52% (33.65%, 35.31%) |
| **10-word registration memory** | | | | |
| **Score** | **MVPA (95%CI)** | **LPA (95%CI)** | **SB (95%CI)** | **Sleep (95%CI)** |
| 0 | 1.92% (1.50%, 2.44%) | 14.71% (11.81%, 18.10%) | 47.78% (40.95%, 54.16%) | 35.59% (34.65%, 36.43%) |
| 1 | 2.04% (1.64%, 2.53%) | 15.18% (12.51%, 18.25%) | 47.66% (41.65%, 53.29%) | 35.12% (34.27%, 35.89%) |
| 2 | 2.18% (1.79%, 2.64%) | 15.66% (13.18%, 18.46%) | 47.51% (42.21%, 52.51%) | 34.64% (33.88%, 35.35%) |
| 3 | 2.32% (1.94%, 2.77%) | 16.15% (13.82%, 18.75%) | 47.36% (42.59%, 51.86%) | 34.17% (33.45%, 34.83%) |
| 4 | 2.47% (2.09%, 2.93%) | 16.65% (14.39%, 19.15%) | 47.19% (42.75%, 51.39%) | 33.69% (33.00%, 34.32%) |
| 5 | 2.64% (2.22%, 3.11%) | 17.16% (14.86%, 19.69%) | 47.00% (42.67%, 51.11%) | 33.20% (32.51%, 33.85%) |
| 6 | 2.81% (2.35%, 3.34%) | 17.68% (15.23%, 20.38%) | 46.80% (42.34%, 51.02%) | 32.71% (31.97%, 33.40%) |
| 7 | 2.99% (2.46%, 3.61%) | 18.21% (15.50%, 21.21%) | 46.59% (41.79%, 51.08%) | 32.22% (31.39%, 32.98%) |
| 8 | 3.18% (2.56%, 3.93%) | 18.75% (15.68%, 22.17%) | 46.35% (41.09%, 51.24%) | 31.72% (30.78%, 32.58%) |
| 9 | 3.38% (2.65%, 4.29%) | 19.29% (15.81%, 23.22%) | 46.11% (40.28%, 51.47%) | 31.22% (30.13%, 32.20%) |
| 10 | 3.59% (2.73%, 4.69%) | 19.85% (15.88%, 24.36%) | 45.84% (39.39%, 51.71%) | 30.71% (29.46%, 31.83%) |
| **10-word recall memory** | | | | |
| **Score** | **MVPA (95%CI)** | **LPA (95%CI)** | **SB (95%CI)** | **Sleep (95%CI)** |
| 0 | 2.23% (1.85%, 2.68%) | 15.97% (13.58%, 18.65%) | 47.48% (42.51%, 52.18%) | 34.31% (33.58%, 34.99%) |
| 1 | 2.36% (1.98%, 2.80%) | 16.35% (14.05%, 18.90%) | 47.33% (42.72%, 51.71%) | 33.96% (33.26%, 34.61%) |
| 2 | 2.49% (2.10%, 2.94%) | 16.74% (14.47%, 19.23%) | 47.17% (42.77%, 51.34%) | 33.60% (32.91%, 34.24%) |
| 3 | 2.63% (2.22%, 3.11%) | 17.13% (14.83%, 19.65%) | 47.01% (42.67%, 51.11%) | 33.24% (32.54%, 33.88%) |
| 4 | 2.78% (2.33%, 3.30%) | 17.52% (15.13%, 20.16%) | 46.83% (42.42%, 50.99%) | 32.87% (32.15%, 33.55%) |
| 5 | 2.93% (2.43%, 3.51%) | 17.92% (15.35%, 20.76%) | 46.64% (42.02%, 50.99%) | 32.51% (31.72%, 33.23%) |
| 6 | 3.09% (2.53%, 3.76%) | 18.33% (15.52%, 21.45%) | 46.44% (41.51%, 51.06%) | 32.14% (31.27%, 32.93%) |
| 7 | 3.26% (2.62%, 4.04%) | 19.58% (15.77%, 23.89%) | 45.79% (39.51%, 51.52%) | 31.01% (29.79%, 32.09%) |
| 8 | 3.82% (2.85%, 5.07%) | 18.74% (15.64%, 22.21%) | 46.23% (40.90%, 51.19%) | 31.76% (30.80%, 32.64%) |
| 9 | 3.44% (2.70%, 4.35%) | 19.16% (15.72%, 23.02%) | 46.02% (40.23%, 51.35%) | 31.39% (30.30%, 32.37%) |
| 10 | 3.63% (2.78%, 4.69%) | 20.00% (15.80%, 24.79%) | 45.55% (38.75%, 51.70%) | 30.63% (29.26%, 31.83%) |
|  | **Verbal fluency test** | | | |
| **Words (n)** | **MVPA (95%CI)** | **LPA (95%CI)** | **SB (95%CI)** | **Sleep (95%CI)** |
| 0 | 1.62% (1.27%, 2.07%) | 15.76% (12.65%, 19.38%) | 49.19% (42.53%, 55.32%) | 33.43% (32.54%, 34.25%) |
| 10 | 2.05% (1.69%, 2.47%) | 16.38% (13.87%, 19.18%) | 48.15% (43.13%, 52.87%) | 33.43% (32.69%, 34.11%) |
| 20 | 2.58% (2.18%, 3.05%) | 16.99% (14.72%, 19.48%) | 47.06% (42.74%, 51.15%) | 33.37% (32.67%, 34.03%) |
| 30 | 3.25% (2.68%, 3.92%) | 17.59% (14.98%, 20.48%) | 45.91% (41.09%, 50.45%) | 33.25% (32.37%, 34.06%) |
| 40 | 4.08% (3.18%, 5.19%) | 18.17% (14.79%, 21.99%) | 44.69% (38.57%, 50.36%) | 33.06% (31.79%, 34.19%) |
| 50 | 5.11% (3.70%, 6.94%) | 18.73% (14.40%, 23.72%) | 43.38% (35.67%, 50.41%) | 32.78% (30.93%, 34.37%) |
| 60 | 6.38% (4.26%, 9.30%) | 19.23% (13.91%, 25.48%) | 41.98% (32.65%, 50.32%) | 32.41% (29.77%, 34.55%) |

**Table S3 Adjusted predictions for 24h activity behaviour across gender**

|  | **Male** | | | | **Female** | | | |
| --- | --- | --- | --- | --- | --- | --- | --- | --- |
|  | **Self-perceived memory** | | | | | | | |
| **Level** | **MVPA (95%CI)** | **LPA (95%CI)** | **SB (95%CI)** | **Sleep (95%CI)** | **MVPA (95%CI)** | **LPA (95%CI)** | **SB (95%CI)** | **Sleep (95%CI)** |
| Poor | 2.55% (2.17%, 2.99%) | 16.68% (14.35%, 19.27%) | 46.34% (42.07%, 50.37%) | 34.43% (33.49%, 35.33%) | 3.16% (2.75%, 3.62%) | 19.84% (17.91%, 21.90%) | 44.97% (41.75%, 48.06%) | 32.04% (31.57%, 32.47%) |
| Fair | 2.38% (2.10%, 2.70%) | 16.45% (14.64%, 18.42%) | 46.60% (43.29%, 49.78%) | 34.56% (33.85%, 35.25%) | 3.06% (2.74%, 3.41%) | 20.26% (18.67%, 21.92%) | 43.73% (41.17%, 46.22%) | 32.96% (32.58%, 33.32%) |
| Good | 2.23% (2.01%, 2.46%) | 16.22% (14.74%, 17.80%) | 46.87% (44.15%, 49.49%) | 34.68% (34.12%, 35.24%) | 2.95% (2.68%, 3.25%) | 20.67% (19.26%, 22.13%) | 42.50% (40.26%, 44.69%) | 33.88% (33.54%, 34.21%) |
| Very good | 2.08% (1.88%, 2.30%) | 15.99% (14.53%, 17.56%) | 47.12% (44.38%, 49.77%) | 34.80% (34.24%, 35.35%) | 2.85% (2.57%, 3.16%) | 21.07% (19.56%, 22.64%) | 41.27% (38.90%, 43.60%) | 34.80% (34.43%, 35.16%) |
| Excellent | 1.94% (1.71%, 2.20%) | 15.77% (14.00%, 17.68%) | 47.37% (43.98%, 50.62%) | 34.92% (34.24%, 35.58%) | 2.76% (2.43%, 3.12%) | 21.46% (19.61%, 23.40%) | 40.06% (37.18%, 42.86%) | 35.73% (35.26%, 36.17%) |
|  | **10-word registration memory** | | | | | | | |
| **Score** | **MVPA (95%CI)** | **LPA (95%CI)** | **SB (95%CI)** | **Sleep (95%CI)** | **MVPA (95%CI)** | **LPA (95%CI)** | **SB (95%CI)** | **Sleep (95%CI)** |
| 0 | 1.85% (1.65%, 2.07%) | 15.24% (13.67%, 16.93%) | 47.49% (44.36%, 50.49%) | 35.43% (34.82%, 36.02%) | 2.48% (2.22%, 2.77%) | 19.18% (17.63%, 20.82%) | 42.80% (40.07%, 45.46%) | 35.54% (35.17%, 35.89%) |
| 1 | 1.96% (1.76%, 2.17%) | 15.55% (14.08%, 17.13%) | 47.31% (44.46%, 50.06%) | 35.18% (34.61%, 35.73%) | 2.61% (2.36%, 2.90%) | 19.66% (18.20%, 21.20%) | 42.64% (40.15%, 45.07%) | 35.08% (34.73%, 35.41%) |
| 2 | 2.07% (1.87%, 2.28%) | 15.87% (14.45%, 17.39%) | 47.13% (44.45%, 49.72%) | 34.93% (34.38%, 35.46%) | 2.76% (2.50%, 3.04%) | 20.15% (18.74%, 21.63%) | 42.47% (40.15%, 44.74%) | 34.62% (34.28%, 34.94%) |
| 3 | 2.18% (1.98%, 2.41%) | 16.20% (14.76%, 17.73%) | 46.94% (44.31%, 49.49%) | 34.68% (34.13%, 35.21%) | 2.90% (2.64%, 3.20%) | 20.65% (19.25%, 22.11%) | 42.29% (40.06%, 44.48%) | 34.16% (33.82%, 34.47%) |
| 4 | 2.31% (2.08%, 2.55%) | 16.52% (15.02%, 18.13%) | 46.75% (44.06%, 49.35%) | 34.42% (33.85%, 34.98%) | 3.06% (2.77%, 3.37%) | 21.15% (19.71%, 22.65%) | 42.10% (39.88%, 44.28%) | 33.69% (33.34%, 34.02%) |
| 5 | 2.43% (2.18%, 2.71%) | 16.86% (15.23%, 18.60%) | 46.55% (43.69%, 49.30%) | 34.16% (33.54%, 34.76%) | 3.22% (2.91%, 3.57%) | 21.66% (20.13%, 23.25%) | 41.90% (39.61%, 44.14%) | 33.22% (32.85%, 33.57%) |
| 6 | 2.57% (2.28%, 2.89%) | 17.19% (15.39%, 19.14%) | 46.34% (43.24%, 49.32%) | 33.89% (33.20%, 34.56%) | 3.39% (3.04%, 3.78%) | 22.17% (20.51%, 23.90%) | 41.69% (39.26%, 44.06%) | 32.75% (32.34%, 33.13%) |
| 7 | 2.71% (2.37%, 3.10%) | 17.53% (15.51%, 19.73%) | 46.13% (42.71%, 49.38%) | 33.63% (32.85%, 34.37%) | 3.57% (3.16%, 4.02%) | 22.69% (20.85%, 24.61%) | 41.47% (38.85%, 44.01%) | 32.27% (31.81%, 32.70%) |
| 8 | 2.86% (2.46%, 3.32%) | 17.88% (15.61%, 20.35%) | 45.91% (42.15%, 49.47%) | 33.35% (32.48%, 34.19%) | 3.76% (3.29%, 4.29%) | 23.21% (21.17%, 25.35%) | 41.24% (38.40%, 43.98%) | 31.79% (31.28%, 32.27%) |
| 9 | 3.02% (2.56%, 3.55%) | 18.22% (15.69%, 21.02%) | 45.68% (41.54%, 49.57%) | 33.08% (32.09%, 34.02%) | 3.95% (3.41%, 4.57%) | 23.74% (21.47%, 26.13%) | 40.99% (37.91%, 43.96%) | 31.31% (30.73%, 31.85%) |
| 10 | 3.19% (2.65%, 3.81%) | 18.57% (15.75%, 21.71%) | 45.44% (40.92%, 49.67%) | 32.80% (31.69%, 33.85%) | 4.16% (3.53%, 4.88%) | 24.28% (21.75%, 26.94%) | 40.74% (37.40%, 43.93%) | 30.83% (30.18%, 31.43%) |
|  | **10-word recall memory** | | | | | | | |
| **Score** | **MVPA (95%CI)** | **LPA (95%CI)** | **SB (95%CI)** | **Sleep (95%CI)** | **MVPA (95%CI)** | **LPA (95%CI)** | **SB (95%CI)** | **Sleep (95%CI)** |
| 0 | 1.81% (1.56%, 2.11%) | 13.97% (12.02%, 16.14%) | 46.89% (42.47%, 51.13%) | 37.32% (36.49%, 38.13%) | 1.96% (1.68%, 2.27%) | 17.77% (15.80%, 19.89%) | 43.64% (39.81%, 47.36%) | 36.63% (36.18%, 37.04%) |
| 1 | 1.88% (1.65%, 2.15%) | 14.41% (12.65%, 16.35%) | 46.94% (43.11%, 50.61%) | 36.77% (36.03%, 37.48%) | 2.12% (1.86%, 2.42%) | 18.34% (16.54%, 20.25%) | 43.40% (40.06%, 46.65%) | 36.14% (35.72%, 36.52%) |
| 2 | 1.96% (1.74%, 2.20%) | 14.87% (13.26%, 16.61%) | 46.97% (43.65%, 50.17%) | 36.21% (35.56%, 36.84%) | 2.30% (2.04%, 2.59%) | 18.92% (17.28%, 20.64%) | 43.15% (40.23%, 45.99%) | 35.64% (35.26%, 35.99%) |
| 3 | 2.03% (1.82%, 2.25%) | 15.33% (13.85%, 16.93%) | 46.99% (44.06%, 49.82%) | 35.65% (35.07%, 36.22%) | 2.49% (2.24%, 2.77%) | 19.50% (18.00%, 21.08%) | 42.87% (40.30%, 45.39%) | 35.13% (34.78%, 35.46%) |
| 4 | 2.10% (1.90%, 2.32%) | 15.81% (14.39%, 17.33%) | 47.00% (44.30%, 49.60%) | 35.09% (34.54%, 35.62%) | 2.70% (2.44%, 2.98%) | 20.10% (18.68%, 21.58%) | 42.58% (40.25%, 44.87%) | 34.62% (34.28%, 34.94%) |
| 5 | 2.18% (1.97%, 2.41%) | 16.30% (14.85%, 17.84%) | 46.99% (44.35%, 49.54%) | 34.53% (33.99%, 35.06%) | 2.92% (2.65%, 3.21%) | 20.71% (19.31%, 22.17%) | 42.28% (40.05%, 44.45%) | 34.10% (33.76%, 34.42%) |
| 6 | 2.26% (2.03%, 2.51%) | 16.79% (15.23%, 18.47%) | 46.97% (44.21%, 49.63%) | 33.97% (33.39%, 34.53%) | 3.16% (2.86%, 3.48%) | 21.32% (19.86%, 22.85%) | 41.95% (39.71%, 44.14%) | 33.57% (33.21%, 33.91%) |
| 7 | 2.34% (2.08%, 2.63%) | 17.30% (15.53%, 19.21%) | 46.94% (43.92%, 49.84%) | 33.41% (32.76%, 34.04%) | 3.42% (3.07%, 3.80%) | 21.94% (20.34%, 23.62%) | 41.61% (39.24%, 43.92%) | 33.03% (32.63%, 33.41%) |
| 8 | 2.43% (2.12%, 2.77%) | 17.82% (15.77%, 20.05%) | 46.90% (43.51%, 50.12%) | 32.85% (32.11%, 33.56%) | 3.69% (3.28%, 4.15%) | 22.57% (20.77%, 24.46%) | 41.25% (38.66%, 43.77%) | 32.49% (32.02%, 32.92%) |
| 9 | 2.52% (2.15%, 2.93%) | 18.35% (15.97%, 20.96%) | 46.84% (43.02%, 50.44%) | 32.29% (31.44%, 33.11%) | 3.99% (3.48%, 4.55%) | 23.21% (21.15%, 25.37%) | 40.87% (38.01%, 43.64%) | 31.93% (31.39%, 32.43%) |
| 10 | 2.61% (2.18%, 3.10%) | 18.89% (16.14%, 21.93%) | 46.78% (42.49%, 50.77%) | 31.73% (30.76%, 32.66%) | 4.30% (3.69%, 5.00%) | 23.85% (21.49%, 26.32%) | 40.48% (37.30%, 43.53%) | 31.37% (30.74%, 31.96%) |
|  | **Verbal fluency test** | | | | | | | |
| **Words (n)** | **MVPA (95%CI)** | **LPA (95%CI)** | **SB (95%CI)** | **Sleep (95%CI)** | **MVPA (95%CI)** | **LPA (95%CI)** | **SB (95%CI)** | **Sleep (95%CI)** |
| 0 | 1.34% (1.15%, 1.57%) | 14.48% (12.41%, 16.80%) | 48.46% (44.00%, 52.69%) | 35.72% (34.91%, 36.50%) | 1.85% (1.59%, 2.13%) | 19.73% (17.64%, 21.97%) | 44.95% (41.42%, 48.36%) | 33.47% (33.04%, 33.88%) |
| 10 | 1.70% (1.51%, 1.90%) | 15.28% (13.67%, 17.03%) | 47.76% (44.57%, 50.83%) | 35.26% (34.64%, 35.86%) | 2.32% (2.08%, 2.59%) | 20.24% (18.63%, 21.93%) | 43.65% (41.02%, 46.20%) | 33.79% (33.43%, 34.14%) |
| 20 | 2.15% (1.95%, 2.36%) | 16.10% (14.68%, 17.62%) | 47.00% (44.38%, 49.54%) | 34.75% (34.20%, 35.28%) | 2.91% (2.65%, 3.20%) | 20.73% (19.33%, 22.19%) | 42.30% (40.08%, 44.47%) | 34.06% (33.71%, 34.39%) |
| 30 | 2.71% (2.41%, 3.03%) | 16.93% (15.21%, 18.78%) | 46.17% (43.17%, 49.06%) | 34.19% (33.51%, 34.85%) | 3.65% (3.27%, 4.06%) | 21.19% (19.59%, 22.85%) | 40.91% (38.44%, 43.33%) | 34.25% (33.80%, 34.67%) |
| 40 | 3.41% (2.91%, 3.98%) | 17.77% (15.39%, 20.38%) | 45.26% (41.28%, 49.02%) | 33.56% (32.58%, 34.50%) | 4.56% (3.95%, 5.25%) | 21.61% (19.53%, 23.79%) | 39.47% (36.30%, 42.55%) | 34.36% (33.70%, 34.98%) |
| 50 | 4.28% (3.46%, 5.24%) | 18.60% (15.41%, 22.18%) | 44.25% (39.12%, 49.02%) | 32.87% (31.46%, 34.19%) | 5.68% (4.72%, 6.81%) | 21.97% (19.30%, 24.79%) | 37.97% (33.95%, 41.86%) | 34.38% (33.38%, 35.27%) |
| 60 | 5.35% (4.09%, 6.92%) | 19.42% (15.35%, 24.05%) | 43.14% (36.84%, 48.86%) | 32.09% (30.15%, 33.86%) | 7.06% (5.59%, 8.83%) | 22.26% (18.99%, 25.74%) | 36.41% (31.53%, 41.10%) | 34.27% (32.83%, 35.53%) |

**Figures**


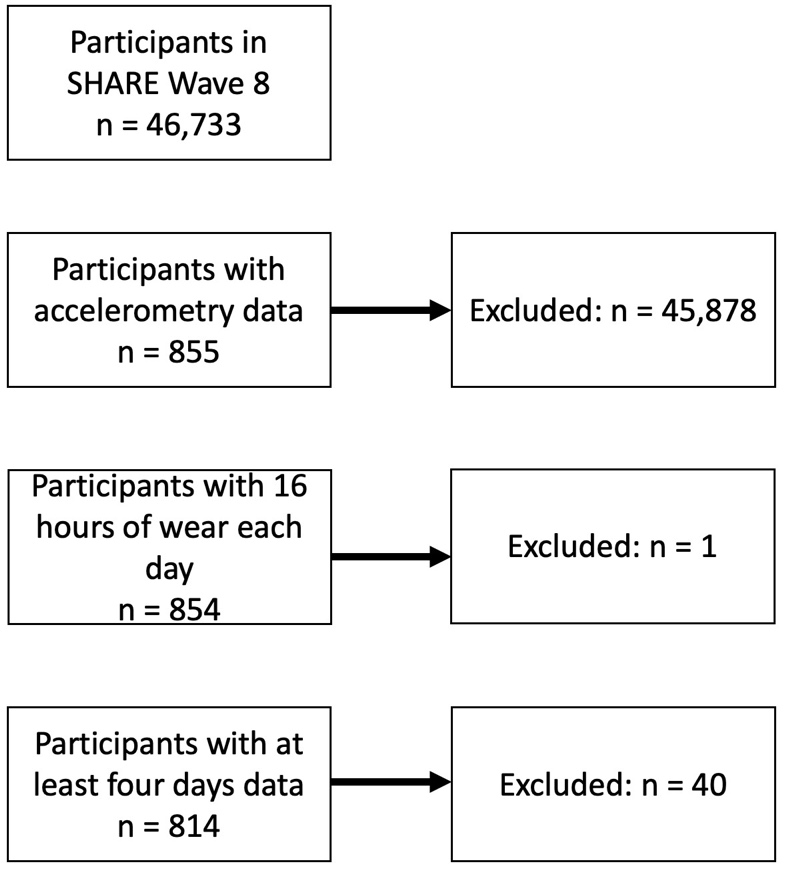


**Figure S1 Participants flowchart**


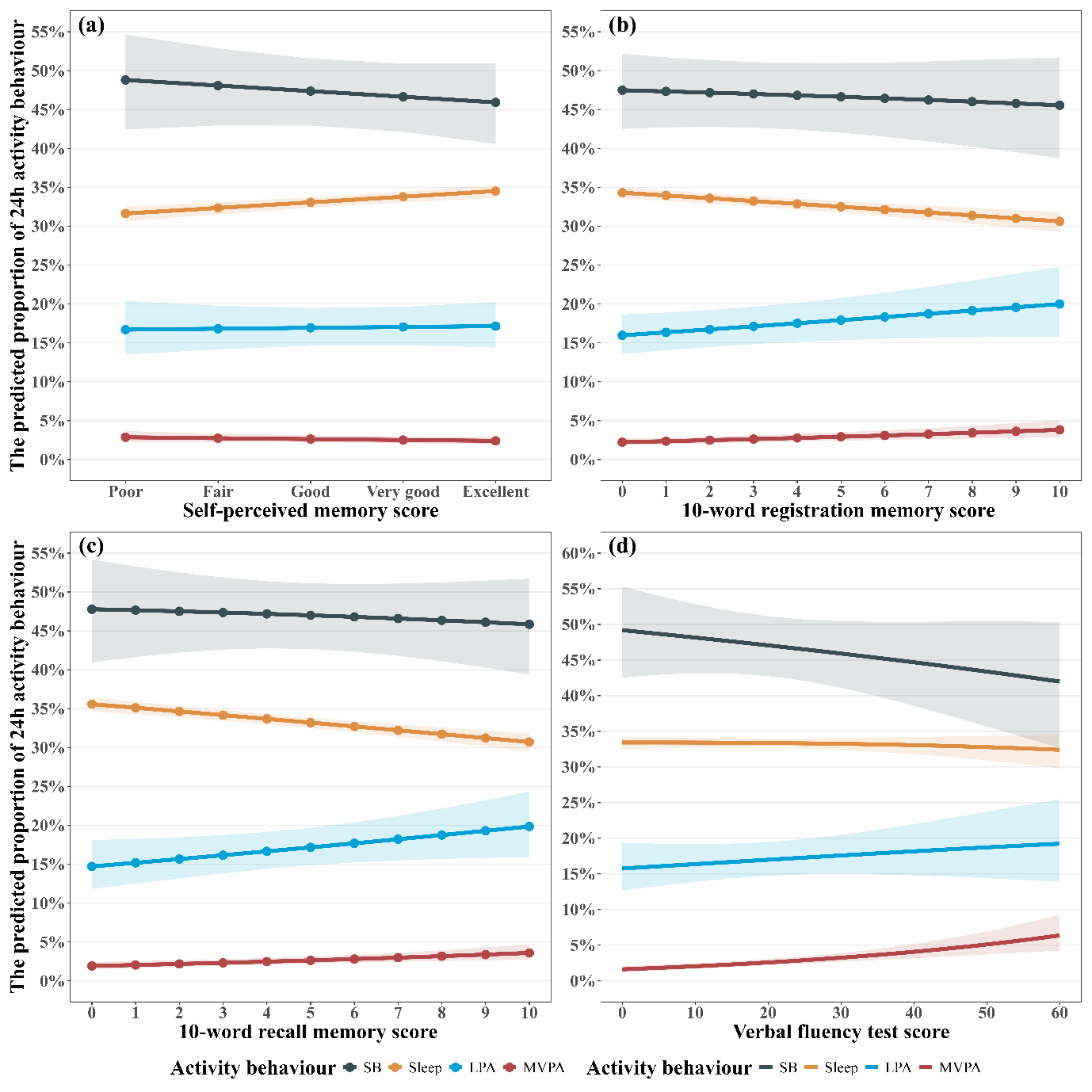


**Figure S2 Adjusted predictions of 24h activity behaviour**


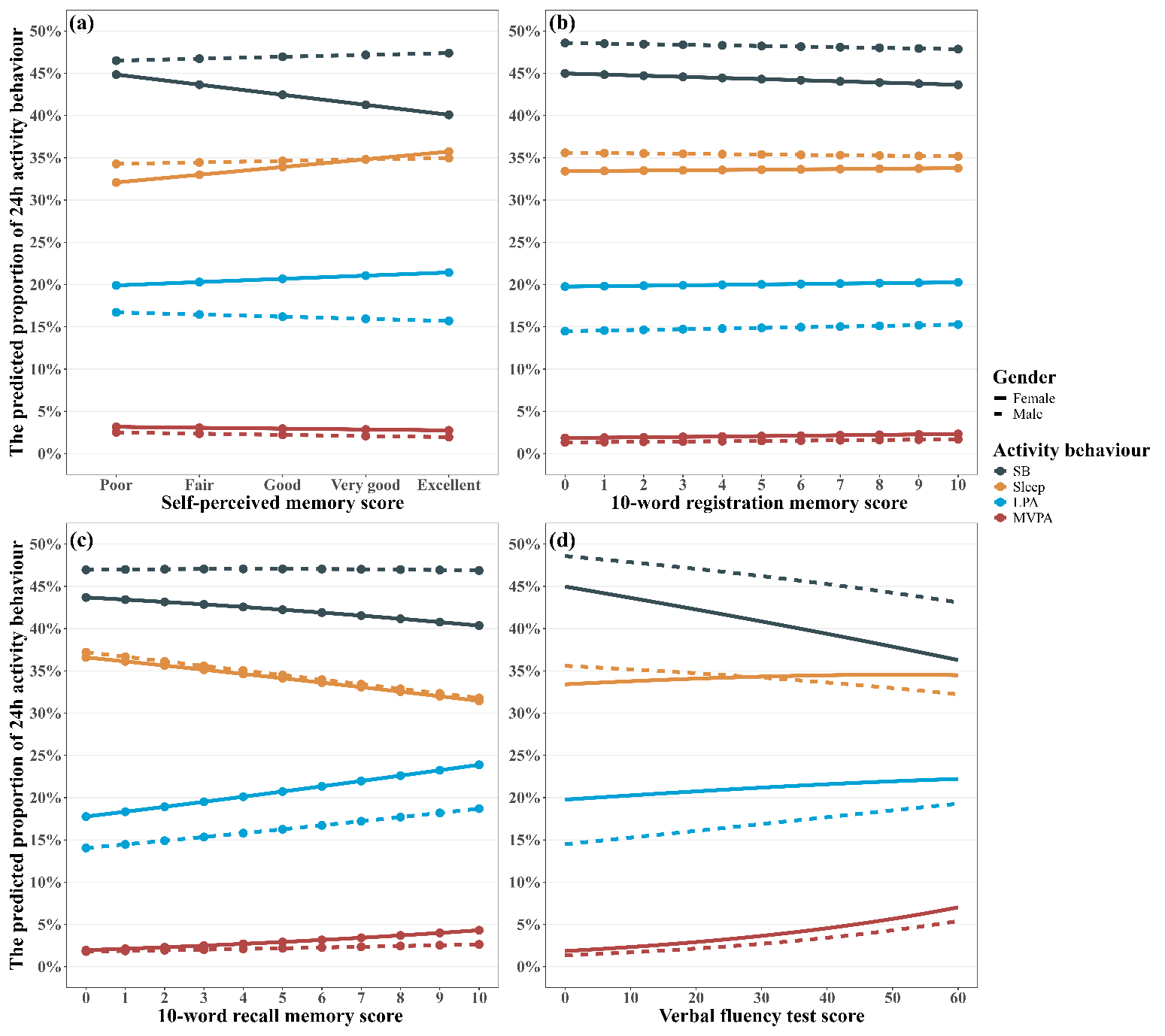


**Figure S3 Adjusted predictions of 24h activity behaviour across sex**
